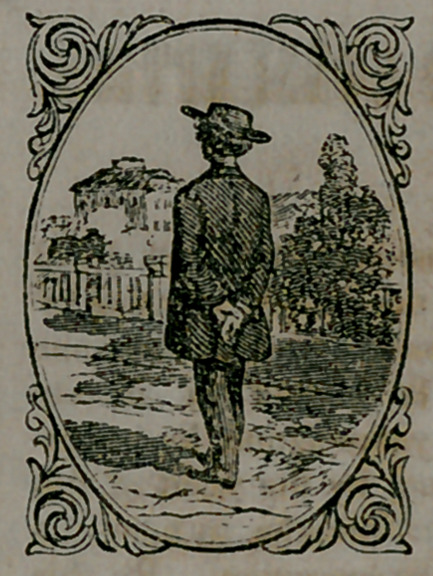# An Erect Position Adverse to Consumption

**Published:** 1869-08

**Authors:** 


### AN ERECT POSITION ADVERSE TO CONSUMPTION.

Who does not shrink with dread and fear at the simple mention of '*Consumption?*' It does not come suddenly. It begins in remote months and years agone, by imperfect breathing; by the want of frequent and *full* breaths, to keep the lungs in active operation. By this neglect, in time, the lungs swell out from a quarter to one third *less* than they ought to do; consequently, the breast flattens, the shoulders bend forward and inward, and we have the round or high shoulder, so ominous in the doctor's eye.

As consumptives *always* bend forward, and as men in high health, *candidates for aldermanic honors,* sit and walk and stand erect—*physically!* the erect position must be antagonistic to consumption, and consequently, such a position should be cultivated, sedulously cultivated, in every manner practicable; cultivated by all, not only by men, but by women and children.

No place is so well adapted to secure an *erect* locomotion as a large city; the necessity is ever present for holding up the head. Instead of giving all sorts of rules about turning out the toes, and straightening up the body, and holding the shoulders back, all of which are impracticable to the many, because soon forgotten, or of a feeling of awkwardness and discomfort which procures a willing omission; all that is necessary, to secure the object, is to *hold up the head and move on!* letting the toes and shoulders take care of themselves. Walk with the chin but slightly above a horizontal line, or with your eyes directed to things a little higher than your head. In this way you walk properly, pleasurably, and without any feeling of restraint or awkwardness.

If you wish to be aided in securing this habitual carriage of body, accustom yourself, while walking, to carry the hands behind you, one grasping the opposite wrist. Englishmen are admired the world over for their full chests, and broad shoulders, and sturdy frames, and manly bearing. This position of body is a favorite with them, in the simple promenade, in the garden or gallery, in attending ladies along a crowded street, in standing on the street, or in places of public worship.

Our young men seem to be in elysium when they can walk arm-in-arm with their divinities. Now, young gentlemen, you will be hooked on quite soon enough, without anticipating your captivity. While you are free, *walk right* in ALL WAYS; and when you are able, get a *manly* carriage; take our word for it, that it is the best way in the world to *secure* the affectionate respect of the woman you marry. Did you ever know any girl worth having, who could or would wed a man, who mopes about with his eyes on the ground, making of his whole body the segment of a circle bent the wrong way? Assuredly, a woman of strong points, of striking characteristics, admires, beyond a handsome face, the whole carriage of a man. Erectness, being the representative of courage and daring, is that which makes a '*man of presence*' in the hour of impending danger or peril."

### Walking or Sleeping, with the Mouth open.

There is one rule which should be strictly observed by all in taking exercise by walking—as the very best form in which it can be taken by both the young and the able-bodied of all ages—and that is, *never to allow the action of respiration or breathing to be carried on through the mouth.* The nasal passages are clearly the medium through which respiration was, by our Creator, designed to be carried on. "God breathed into man's nostrils the breath of life," previous to his becoming a living creature.

The difference in the exhaustion of strength by a long walk with the mouth firmly and resolutely closed, and respiration carried on through the nostrils instead of through the mouth, can not be conceived as possible by those who have never tried the experiment. Indeed, this mischievous and really unnatural habit of carrying on the work of inspiration and expiration through the mouth, instead of through the nasal passages, is the true origin of almost all diseases of the throat and lungs, *bronchitis, congestion, asthma,* and even *consumption* itself.

That excessive perspiration to which some individuals are so liable in tneir sleep, and which is so weakening to the body, is solely the effect of such persons sleeping with their mouths unclosed. And the same exhaustive results arise to the animal system from walking with the mouth open, instead of—when not engaged in conversation—preserving the lips in a state of firm but quiet compression. Children should never be allowed to sleep, stand, or walk, with their mouths open; for, besides the vacant appearance it gives to the countenance, it sometimes causes *coughs, colds,* and *sore throats.*

## Figures and Tables

**Figure f1:**
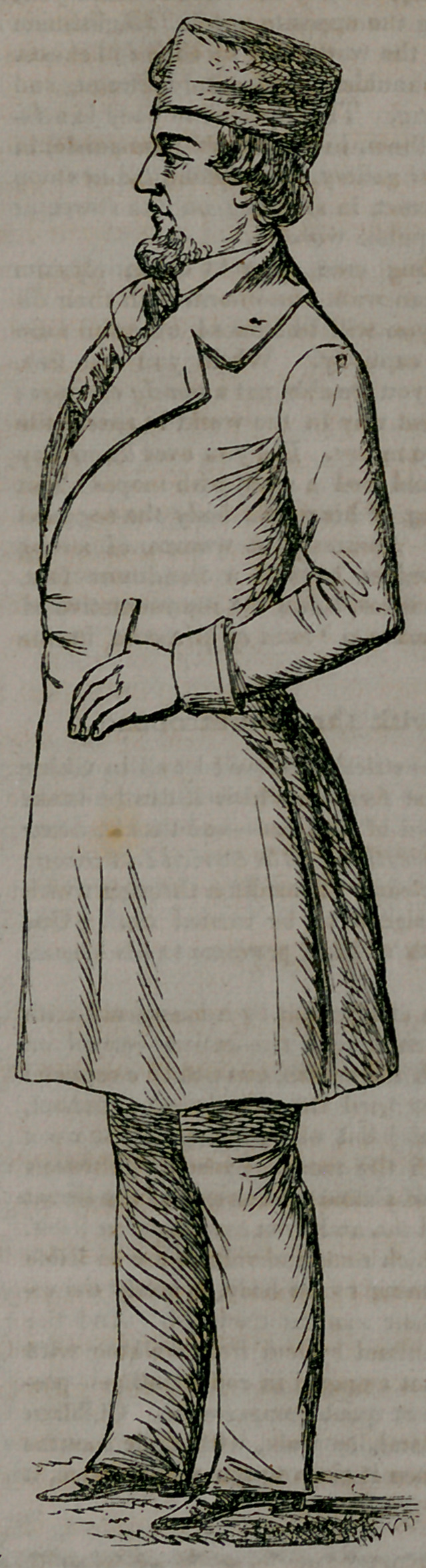


**Figure f2:**